# Determinants of road traffic injuries in Iranian children; results from a National Representative Demographic- Health Survey 2010

**DOI:** 10.1186/s12887-020-02127-4

**Published:** 2020-05-19

**Authors:** Hesam Ghiasvand, Payam Roshanfekr, Delaram Ali, Hossein Malekafzali Ardakani, Amanda N. Stephens, Bahram Armoon

**Affiliations:** 1 Health Management and Economics Research Center, Iran University of Medical Sciences, Tehran, Iran; 2 Health Economics Group, Medical School, Saint Luke’s Campus, University of Exeter, Exeter, UK; 3 Social Determinants of Health Research Center, University of Social Welfare and Rehabilitation, Tehran, Iran; 4 School of Public Health, Tehran University of Medical Sciences, Tehran, Iran; 5 Monash University Accident Research Centre, Monash University, Clayton, Australia; 6 Social Determinants of Health Research Center, Saveh University of Medical Sciences, Saveh, Iran; 7 School of Nursing and Midwifery, Saveh University of Medical Sciences, Tehran-Saveh freeway, Kaveh Industrial Estate company, Saveh, 3914334911 Iran

**Keywords:** Road traffic injuries, Demographic and health survey, Iranian multiple Indicator demographic and health survey, Socio-economic factors, Iranian children

## Abstract

**Background:**

Road Traffic Injuries (RTIs) are a leading cause of disabilities and mortalities in Iran. The occurrence of RTIs among children is increasing. This study aims are to assess RTIs among Iranian children and to determine the main socio-economics determinants.

**Methods:**

The National Institute of Health Research (NIHR) in collaboration with the Iran Ministry of Health (MoH) conducted a nationwide survey: The Multiple Indicator Demographic and Health Survey 2010 (IrMIDHS 2010). The Survey was undertaken by Medical Universities in Iran. Based on multistage clustered randomized sampling, 30,960 households were included in the survey. We performed a multivariate logistic regression to determine the main socio-economic factors associated with RTIs among children.

**Results:**

Approximately 0.9% of the children received RTIs in 2010. Main socio-economics contributors to RTIs involving Iranian children included household size (Adjusted OR: 1.06 (CI 95% 1.01, 1.14), sex (Adjusted OR_female_: 0.38 (CI 95% 0.29, 0.50), living with both parents (Adjusted OR: 0.55 (CI 95% 0.13, 0.95), being in the 2nd (Adjusted OR: 0.81 (CI 95%: 0.60, 0.90) or 4th income quartile (Adjusted OR: 0.13 (CI 95%: 0.02, 0.92) rather than the 1st income quartile, being aged five to nine (Adjusted OR: 1.39 (CI 95%: 1.10, 2.10), or aged 15 to 18 (Adjusted OR: 2.94 (CI 95%: 2.07, 4.97), and residency in a non- owned or non-tenancy house (Adjusted OR: 0.42 (CI 95%: 0.23 0.74).

**Conclusions:**

Children need safe places for playing and doing their daily activities. Policy and regulation development aimed at protecting children from road traffic injuries needs to take into consideration the socio-economic factors associated with risk of road traffic injury among children.

## Introduction

### Background

Road Traffic Injuries (RTIs) are one of the leading causes of disability or mortality in children [1]. For example, in India, the rate of injuries and deaths per 100,000 children is 40 and 11, respectively [2]. In South Asia and Africa, this rate is 7.4 and 19.9 per 100,000 children, respectively [3]. The mortality and disabilities attributable to road traffic crashes in Iran is considerably higher. In 2016 alone, road traffic injuries were linked to 35.6 (29.64–43.33) deaths per 100,000 children [4].

The Iran Legal Medicine Organization (LMO) reported a total of 20,068 road traffic deaths and 367,451 road traffic injuries during 2017. Children aged 17 years or younger constituted a notable percentage of those injured in Iran, representing approximately 14% of those receiving RTIs [5–8].

Driver, vehicle and environmental factors contribute to road traffic collisions. These include, unsafe vehicles, low quality control for produced vehicles, poor quality roads, risky driving behaviors and poorly developed and implemented preventive regulations are among the main reasons for road traffic collisions in Iran. In addition, there is also an increase in vehicles on the road. In recent years, vehicles have become more affordable and accessible in Iran. By way of example, according to the reports of the Iran Vehicle Manufacturers Association, the total number of automobiles produced in Iran increased from 924,874 in March 2015 to 1,245,691 in March 2016 [9, 10].

Over the past decade, several policies and regulations aimed at reducing RTIs have been implemented in Iran. This has led to improvements in driving behavior and an associated decrease in the number of road traffic crashes. However, Iran continues to have a high rate of RTIs compared to other countries. As such, RTIs remain a major public health concern [11, 12].

Road safety is a priority area in Iran. Previous National Development Plans by the Ministry of Roads and Urban Development and Statistical Centre of Iran stated that a nationwide database for registering RTIs in the country needs to be established. Renewing the transportation system and targeting a 10% reduction in fatalities (between 2014 and 2015) were among the priorities of the Iran government for reducing RTIs. Furthermore, based on the Plan articles, medical centers and hospitals are now obliged by the Government to provide services and care for those injured in road traffic crashes; without restrictions or financial cost to the patient [13].

The policies and regulations discussed above may lead to favorable outcomes in the near future. Nevertheless, more is needed to protect children from RTIs. Children are involved in a considerable number of RTIs; both globally and in Iran. Indeed, UNICEF has reported RTIs are a major cause of death among children, particularly those aged 5 years and younger. In Iran, a recent study has shown that a large number of RTI victims (*n* = 3578) were children and adolescents under 19 years of age [14]. Children have the right to play in safe places and the current transportation system should not place them at undue risk [15, 16]. Therefore, tackling this problem may be the first step in understanding the socio-economic factors associated with it.

### Objective

This study aims to examine RTIs among Iranian children and to determine which socio-economic factors are associated with RTI involvement.

## Methods

### Study design

This is a cross-sectional analytical study on the data obtained from the Iran Multiple Indicator Demographic and Health Survey in 2010.

### Setting

The IrMIDHS was primarily based on the Multiple Indicator Cluster Survey 4 (MICS4) developed by United Nations Children’s Fund (UNICEF), and the European regional office of World Health Organization Demographic Health Survey (DHS) in 2001. The National Institute of health research (NIHR) adapted contents of these to specifically meet the needs of Iranian health policy and planners. The IrMIDHS encompasses three main questionnaires that are available in five formats. The questionnaires include three detailed modules for i) households, ii) 15–54-year-old women and iii) children under age 5 years. Details on the development of the Persian version of the questionnaire are available in the published protocol of IrMIDHS [17].

### Participants

The IrMIDHS is a nationwide survey on Iranian rural and urban households. It includes various ages and demographics in the population. For the purpose of the current study, we focused on participants aged 18 years or younger [17].

### Data collection

Household data were collected from one member of each household, who was aware of the households’ information and completed the interview. Women and mothers or child’s caregivers were the main interviewees for children under age 5 years. More details on how the interviews were conducted can be found in the study protocol [17].

### Study size

The sampling framework for conducting IrMIDHS and determining the final sample size derived from a joint collaboration between Statistical center of Iran (SCI) and NIHR [17]. The sampling method was a multiple stratified cluster random. The IrMIDHS data were stratified by rural and urban regions, a conventional sampling method of SCI for conducting the annual Survey on the Urban and Rural Income-Expenditure. The clusters were randomly defined for all districts across the country. Within each cluster, through a predefined systematic sample, 10 households were consecutively selected. The sample size was 3096 households including 909 rural households and 2187 urban households. For the purpose of the current study, we included a population aged 18 or younger. There was a total population of 34,962 eligible children (see the study protocol for more details [17]).

### Quantitative variables

The list and definitions of the variables are:

- Child sex (Male, Female): Self-statement of interviewee member of the household.
- Location of residence (urban/rural): This was defined based on the geographical location of the household at the time the IrMIHDS was conducted. This was defined based on SCI’s definition.
- Children were categorized into five age groups (under 5 years old; 5–9 years old; 10–14 years old and 15–18 years old): Age was recorded based on the statement provided by the child’s mother or caregiver. Age was recorded in monthly units for newborns up to one-year-old and in yearly units for children older than one.
- Household size: This was provided by interviewed member of the household.
- Household income: This was self- statement in accordance one of the following income categories:
   o (under 250,000 IRR^1^ (24 US$);
   o 250,001 IRR (24US$)-750,000 IRR (72.4 US$);
   o 750,001 IRR (72.4 US$)- 1,250,000 IRR (120.65US$);
   o 1,250,001 IRR (120.65US$)-1,750,000 IRR (170 US$); and
   o more than 1,750,000 (170 US$) IRR.
- Home ownership status (owner, rental, other): This was a self-stated variable by the interviewed member of the household.
- Basic health insurance (have or have not): This measures whether the household would access one of the currently available public mandatory health insurance programs in the country. This provides information about whether the child is eligible for cover under one of the public funded current health insurance in the country. The child’s mother or caregiver answered the question.

### Statistical methods

As mentioned above, the IrMIHDS used a stratified cluster sampling design. This implies the weight (proportion) of each cluster (district) on the regression models. We performed the *svyset dist* command in STATA, to address any probable effects on the results. We then performed a bivariate logistic regression to select the final included variables for interested multivariate logistic regression. After this, we used an “Enter” approach to estimate the regression model. The adjusted odd ratios and associated 95% confidence intervals were used to predict the effect of covariates on the probability of exposure to RTIs among children.

### Ethics

The IrMIHDS data were accessed after the permission to do so was granted from the authorized office of the Iran Ministry of Health. This study has the approval of Ethical Committee of the University of Social Welfare and rehabilitation Sciences.

## Results

### Participants

The final regression analysis was conducted on 34,962 children aged 18 or younger. These were all eligibly aged children from a larger sample of participants in the IrMIHDS.

### Descriptive data

Households had an average of five (1.7) members, and the average age of sample of children was 13 (5.6). In addition, 51.6% of the children were male, and 64.6% of them lived in urban regions. The majority of the children (66.6%) lived in houses that were owner-occupied. Approximately 80.4% of the children lived in households that were classified in the first and second income quartiles.

The health insurance coverage for children, as a criterion for availability and accessibility to basic health services, was approximately 80%. The largest age group was the 15–18 age group with almost one third of children in this age range (29.3%). Under 5 years had 26.7% of the sample. In the previous year from which the survey was conducted (2010), approximately 0.9% of the children had been involved in at least one road traffic crash resulting in injury.

Finally, at the time of the survey, a large percentage (93.4%) of the sample lived with both their parents. Table 1 shows, the main descriptive results of the study.

**Table 1:** Descriptive Statistics of Population Characteristics

Variable	Frequency	Percentage (%)	Mean (SD)
Household Size			4.88 (1.7)
Age			13 (5.6)
Gender:			
Male	18,037	51.6	
Female	16,925	48.4	
Residential Area:			
Rural	12,344	35.3	
Urban	22,618	64.7	
Ownership of House:			
Owner	23,290	66.6	
Tenant	7552	21.6	
Other	4120	11.8	
Income Quartiles:			
1st	10,589	33.1	
2nd	15,107	47.3	
3rd	5316	16.6	
4th	943	2.9	
Health Insurance Coverage:			
No	27,916	79.9	
Yes	7030	20.1	
Age Category:			
< 5	9345	26.7	
5–9	6817	19.5	
10–14	8550	24.5	
15–18	10,250	29.3	
RTIs:			
No	34,650	99.1	
Yes	312	0.9	
Having Parental Supervision:			
None of them	103	0.3	
Single parent	2057	6.3	
two parent	30,511	93.4	

### Main results

Table 2 shows the main socio-economics factors predicting the probability of occurrence of RTIs among children.

**Table 2:** Main determinants of RTIs in children

	Crude Odd Ratio	Adjusted Odd Ratio (AOR)	Standard Error	Z statistics	95% Confidence Interval	
Household size	3.52	1.06*	0.04	1.84	1.01	1.14
Residency in Rural Areas	0.15	0.83	0.13	−1.3	0.61	1.11
Female Sex		0.38*	0.06	−6.70	0.29	0.50
Parental Status:						
None of them	1.00	1.00				
Single-parent family	0.95	0.48	0.37	0.94	0.11	0.88
Dual parent family	0.27	0.55*	0.40	−1.42	0.13	0.95
Having Health Insurance	0.54	0.91	0.15	−0.54	0.66	1.13
Income Quartiles:						
1st	1.00	1.00				
2nd	4.21	0.81*	0.12	−1.47	0.60	0.90
3rd	1.25	1.03	0.19	0.17	0.74	1.49
4th	1.35	0.13*	0.13	−2.04	0.02	0.92
Age Category:						
< 5	1.00	1.00				
5–9	3.54	1.39**	0.29	1.57	1.10	2.10
10–14	2.55	0.90	0.20	−0.47	0.58	1.40
15–18	3.65	2.94*	0.53	5.71	2.07	4.97
Home ownership:						
Owner	1.00	1.00				
Tenant	2.12	0.91	0.15	−0.58	0.64	1.27
Other	1.15	0.42*	0.13	−2.85	0.23	0.74
	No. of Observation = 29,761 LR Chi2 (14) = 14,720 Prob. > Chi2 = 0.000 Pseudo R2 = 0.11					

* *P* ≤ 0.05 ** *P* ≤ 0.1

The number of members in a household was positivity associated with the probability of RTI. The results showed that for each increase on this covariate, the odds of RTI were increased by 6% (Adjusted OR: 1.06 (CI 95% 1.01, 1.14) The probability of RTIs was lower for females than for males, with the risk for The risk to females was 62% lower than for males (Adjusted OR: 0.38 (CI 95% 0.29, 0.50). Children who were living with both parents, also had lower odds of RTIs compared to children not living with a parent. Table 2 shows that supervision by both parents is associated with a lower probability of exposure to the RTIs, reducing it by 45% compared to living with no parents (Adjusted OR: 0.55 (CI 95% 0.13, 0.95).

Children in households classified in the second and fourth income quartiles, were less likely to have received RTIs compared to children in first quartile households. Representation by one of these two income quartiles was associated with reductions in the odds of RTIs by approximately 19% (Adjusted OR: 0.81 (CI 95%: 0.60, 0.90) and 87% (Adjusted OR: 0.13 (CI 95%: 0.02, 0.92) respectively.

Children aged five to nine and 15 to 18 had higher odds of being exposed to RTIs compared to children under five. The odds were stronger in older age (15–18) group, where the odds of RTI were almost three-fold (Adjusted OR: 2.94 (CI 95%: 2.07, 4.97) compared to children under five. The odds for children aged five to nine were (Adjusted OR: 1.39 (CI 95%: 1.10, 2.10)). Living in a house that may be occupied without any payments for rent (organizational home, or some other houses that provided by public and governmental authorities), was associated with lower odds of RTIs compared to living in an owner-occupied dwelling. As can be seen in Table 2, children in these households had 58% lower odds (Adjusted OR: 0.42 (CI 95%: 0.23 0.74).

## Discussion

### Principal findings

The main socio-economic factors associated with the probability of RTIs in children were increasing household size, being female, supervision by both parents, living in households classified in the second and fourth income quartiles, being aged between five and nine or 15 to 18, and living in a free of charge non-owned, and or non-rental house.

Our results align with previous research. For example, studies show that living in a household with more members, and being in age groups 5–9 and 15–18 years old are associated with a higher likelihood of RTI among children [18–20]. This is consistent with the results of our study. We also found that the household size and being in some age groups were significantly associated with RTI.

Being female, under supervision of both parents, being in second and fourth income quartiles, or residing in homes that are free of charges or provided by public or non-public providers were associated with a lower likelihood of RTI in children. These findings are consistent with other previous evidence [21, 22].

Compared to the first income quartile, being in second and fourth income quartiles had a protective effect against the risk of RTI among children. However, the explanations for these findings differ for each of level of income. For instance, in the second income quartile the children might have less accessibility to bicycles or motorbikes. It should also be noted that this group of children might be residing within the marginalized regions, having greater exposure to road dangers and other types of risk factors associated with RTI. For example, more frequent interactions with railways or highways near to their residential areas.

However, for children in richer households the story may be different. They enjoy more well-designed environmental conditions, and their entertainment may not be playing in streets. In this group of children, especially in older age groups (e.g., older than 15 years), children may participant in more risky behaviors, such as less supervised, riding bicycles or traveling in other vehicles. The ownership status of families’ housing alongside the income levels were two main economic determinants of RTI in our study. Accordingly, children from households living in free of charge houses are less likely to suffer from RTI. Some studies have found that road traffic injuries are more common in children of poorer households. However, in these studies, the focus was more on post-trauma and post injuries in children and the roles of regional, environmental and parental care were not considered. Our results are inconsistent with the published literature, which suggests that the children in richer households are less likely to be involved in RTIs. One explanation for this inconsistency is that in Iran we should seek other economic proxies to understand the impact of economic factors on the probability of RTIs. These may include households’ wealth, consumption expenditure, or assets. Ranking households based on their income alone, might have some misleading outcomes in interpreting the results [23, 24].

Many of the socio-economic factors associated with RTIs are not modifiable by the households alone. It therefore appears that the reduction or mitigation of RTIs among children requires an interactive engagement between households and public or governmental authorities. This means adopting a public involvement model to address the problem. Within the households, parents and other adult members can advise and guide children about road safety. This may be in regards to the time, places and potential dangers of playing or crossing roads. Members of the households can play a substantial role in increasing road safety awareness to their children.

The authorities can contribute though better infrastructure and the design of children friendly spaces in residential areas. This is one of the principal recommendations that has been raised by the World Health Organization [1]. These places should be secure and keep children away from potential risk of road accidents. Here, local authorities including villages and cities council, play an important role.

The above actions are more challenging in some locations. In particular, areas that are near a railway, public and private transport vehicles or road traffic. Advisory or warning actions need to undertaken, perhaps through the installation of signs alerting drivers to child playing spaces. Using evidence gained in more economically developed countries, such as Canada, Sweden and Japan. may also be useful in setting the agenda for improving road safety for children. In these countries, the legislation has focused mainly on street spaces, school routes, safety parks. World Health Organization develop a guide for establishing and operating Child Friendly Spaces (CFS) which can be useful in building capacity for preventing and improving the safety of children against RTIs [25].

### Strengths and weaknesses of the study

This study analyzed a national survey and provides an understanding of some factors associated with RTIs among children in Iran. The focus of the research was on available and accessible socio-economic data in relation to RTI involvement. The results provide insight for policy makers within the country to develop evidence-based regulations to reduce or mitigate RTIs among children in Iran. More research is warranted to address other relevant and important aspects of RTIs that were not included in this study. These may include a multi-level analysis and region based comparison of RTIs. In addition, analysis of current policies and plans to reduce RTIs among children using a network of stakeholders, may provide more clarifications for future directions.

### Value of the study

RTIs have remained a leading cause of mortality and disability in recent years. However, while the rate of mortality has decreased, disability has increased steadily. This challenge is most concerning for children. The findings of our study highlight some social, environmental and demographic factors that are associated with higher risk of RTIs. Policy makers need to consider these factors when implementing strategies to improve road safety. For example, appropriate local policies for avoiding deaths or disabilities in can consider or target specific road users, based on a child’s gender, their age group, the household’s income levels or size or neighbors and paternal supervision status. Doing this may be beneficial in the reduction of RTIs.

### Unanswered questions and future research

Researchers must also consider social determinants when undertaking further exploration of the determinants of RTIs in children. This approach includes individual and contextual factors, putting them together to provide a clearer picture of the current situation, motivating potential solutions. Because of some limitations in data resources of this study, we focused our present analysis on individual factors and could not provide a multi-level analysis. Future research, if possible, could draw links between the current dataset and other social and health systems databases in Iran to provide additional insights.

## Conclusions

Children need safe places for playing and doing their daily activities. Policy and regulation development aimed at protecting children from road traffic injuries needs to take into consideration the socio-economic factors associated with risk of road traffic injury among children.

## Data Availability

The datasets used and/or analyzed during the current study are available from the corresponding author on reasonable request.

## Abbreviations

IrMIDHS 2010: Iranian Multiple Indicator Demographic and Health Survey 2010
RTA: Road traffic accidents
DALYs: Disability adjusted Life Years
LMO: The Legal Medicine Organization
MoHME: Ministry of Health and Medical Education
MICS: Multiple Indicator Cluster Survey

## Footnotes

1 - 1US$ = 10,360 Iran Rial (IRR) in 2010.
